# The impact of overlapping functional dyspepsia, belching disorders and functional heartburn on anxiety, depression and quality of life of Chinese patients with irritable bowel syndrome

**DOI:** 10.1186/s12876-020-01357-1

**Published:** 2020-07-06

**Authors:** Xin Yao, Yunsheng Yang, Shutian Zhang, Yu Shi, Qian Zhang, Yongjun Wang

**Affiliations:** 1 Department of Gastroenterology, Beijing Friendship Hospital, Capital Medical University, National Clinical Research Center for Digestive Disease, Beijing Digestive Disease Center, Beijing Key Laboratory for Precancerous Lesion of Digestive Disease, No. 95 Yongan Road, Beijing, 100050 China; 2 Department of Gastroenterology, Bethune International Peace Hospital, No. 398 Zhongshanxi Road, Shijiazhuang, 050082 China; 3 Department of Gastroenterology and Hepatology, The First Medical Center, Chinese PLA General Hospital, No. 28 Fuxing Road, Beijing, 100853 China; 4 Clinical Epidemiology and EBM Unit, National Clinical Research Center for Digestive Disease, Beijing Friendship Hospital, Capital Medical University, Beijing, 100050 China

**Keywords:** Irritable bowel syndrome, Psychological factors, Health- related quality of life, Functional dyspepsia, Belching disorders, Functional heartburn

## Abstract

**Background:**

Functional dyspepsia (FD), belching disorders (BD) and functional heartburn (FH) are the three most common upper functional gastrointestinal disorders (FGID) in IBS patients. FD is known to exert deleterious effects on health-related quality of life (HRQoL) and the psychological status of IBS patients; however, the impact of overlapping BD and FH on anxiety, depression and HRQoL of IBS patients remains unknown. This cross-sectional study was conducted to investigate the impact of overlapping FD, BD and FH on anxiety, depression and HRQoL in patients with IBS.

**Methods:**

This study enrolled 319 consecutive outpatients with IBS from 2 tertiary hospitals in Beijing and Shijiazhuang of China. IBS, FD, BD and FH were diagnosed using the Rome III Criteria. Hospital Anxiety and Depression Scale and a 36-item Short-Form Health Survey (SF-36) were used to assess the psychological distress and HRQoL of patients respectively.

**Results:**

Among the 319 patients with IBS, the IBS subtypes were diarrhoea (67%), constipation (16%), unsubtyped (12%) and mixed (5%). These IBS patients were further classified into IBS + FD, IBS + BD/FH (BD and/or FH), IBS + FD + BD/FH, or IBS only according to the patients’ overlapping upper GI symptoms. IBS+FD patients reported higher levels of anxiety than IBS+BD/FH and elevated depression scores than IBS only patients (*P*< 0.05). The latter observation remained consistent after confounder-adjustment. The IBS + FD and IBS + FD + BD/FH groups exhibited statistically significant impairment in most of SF-36 scales, while the IBS + BD/FH group only showed lower HRQoL results in general health, when compared to the IBS only group. Multiple linear regression analysis demonstrated IBS + FD + BD/FH was linked to worse mental, physical and global HRQoL. Furthermore, IBS + FD was a strong predictor of poorer physical and global HRQoL compared to IBS only.

**Conclusions:**

Among the diarrhoea-prevalent IBS patients, those with concomitant FD experienced more psychological distress and demonstrated poorer physical HRQoL. Overlapping FD + BD/FH is a significant predictor of worse mental and physical HRQoL for IBS patients. The impact of concomitant BD and FH on the psychological status and HRQoL of IBS patients was limited. These findings implied that the overlapping upper FGIDs in IBS might be treated distinctively when developing comprehensive management strategies for IBS treatment.

## Background

Irritable bowel syndrome (IBS) is a chronic, often disabling gastrointestinal disorder characterized by abdominal pain or abdominal discomfort associated with a change in bowel habits. The prevalence of IBS diagnosed using the Rome III criteria in the general population has been reported to be 29.2% [1]. Although IBS has been defined and classified as a functional bowel disorder, recent studies involving the general population and patients in clinical settings have demonstrated that there is a high proportion of patients satisfying the diagnostic criteria for both IBS and functional dyspepsia (FD) [2–4]. In the meanwhile, belching disorders (BD) is also prevalent among IBS patients in clinical practice. Our recent multicenter study showed that BD was the second most common upper functional gastrointestinal disorder (FGID) among IBS outpatients based on the Rome III criteria, accounting for 27.1% of IBS patients [5]. The frequent coexistence of BD among IBS subjects was also observed by Park et al. from Korea and Singh P et al. from India [6, 7]. Functional heartburn (FH) was also one of the most commonly seen upper FGIDs in IBS patients [6, 8, 9]. The prevalence of FH ranked the third among upper FGIDs in individuals with IBS based on our investigation [5].

Psychiatric disorders are common in FGIDs, with as many as 67.7 and 47.3% of patients with IBS having measurable anxiety and depression symptoms respectively [7, 10]. The high rates of anxiety and depression were also observed in patients meeting the criteria for FD [8, 10]. Two Korean studies showed depressive mood was significantly related to FD and IBS + FD overlap rather than IBS alone based on Rome III criteria [2, 11]. On the other hand, anxiety and depression are associated with gastrointestinal symptom burden, sleep disturbance and decreased health-related quality of life (HRQoL) [12–15], and may be predisposing factors for FD and IBS [16]. More recent evidence shows that the gut and brain interact bi-directionally in both IBS and FD [17, 18].

A number of out-patients and general population studies suggest that IBS and FD can impair HRQoL, and the impact seems to be on all major variables of quality of life, namely mental, physical, and social domains [19–22]. In addition, patients with FD + IBS tend to have worse quality of life than patients with FD alone and IBS alone [11]. Other data suggested that HRQoL was significantly worse in IBS patients with heartburn than those without. However, neither the concomitant BD or FH in FD + IBS patients nor overlapping BD or FD in IBS patients with heartburn has been take into account in the above two studies.

Although the coexistence of BD and FH among IBS was frequent, the impact of concomitant BD and FH on depression, anxiety and HRQoL of IBS patients is lacking. The aims of this study were to investigate the impact of overlapping upper functional gastrointestinal disorders, including FD, BD and FH, on the psychological problems and quality of life in individuals with IBS.

## Methods

### Study setting and participants

This observational cross-sectional study was conducted in gastroenterology departments of 2 tertiary hospitals in China (Chinese PLA General Hospital in Beijing and Bethune International Peace Hospital in Shijiazhuang) between January 2008 and March 2009.

The consecutive outpatients above 18 years of age in the two hospitals were initially assessed by the gastroenterologist based on Rome III criteria of IBS. All enrolled IBS patients were invited to undergo routine laboratory test, abdominal ultrasound and colonoscopy to rule out organic and metabolic diseases. The patients who were more than 40 years old with upper gastrointestinal symptoms or had a family history of GI malignancy or had one of the alarm symptoms including anemia, GI bleeding, fever, weight loss and dysphasia were also asked to undergo a gastroscopy. The details have been described before [5]. All patients with the following conditions were excluded: (1) diabetes mellitus, hyperthyroidism, hypothyroidism and other metabolic diseases; (2) malignant diseases at any site, liver cirrhosis, advanced chronic kidney disease, heart failure and other severe illnesses; (3) Infectious diseases of the gastrointestinal system caused by bacteria, viruses, parasites, etc.; (4) peptic ulcer, reflux esophagitis, Barrett’s esophagus, inflammatory bowel disease confirmed by gastroscopy and colonoscopy; (5) history of digestive tract surgery;(6) other organic diseases. Then the patients were invited to participate in a face-to-face interview to complete the questionnaires. A total of 367 patients who met the Rome III criteria for IBS were recruited from the 2 hospitals and 319 patients agreed to participate in the study. The study protocol was approved by the Ethics Committees of the two hospitals. Written informed consent was obtained from the patients prior to enrolment.

### Questionnaire

All demographic and symptom data was collected by face-to-face interviews. IBS, FD, BD and FH were diagnosed using the Rome III diagnostic questionnaire for the adult [5]. IBS was further subtyped into IBS-C, IBS-D, IBS-M and IBS-U on Rome III criteria according to the Bristol Stool Form Scale. Symptom overlap was considered to be present when patients with IBS had symptoms compatible with FD, BD and FH in accordance with Rome III criteria.

All IBS patients were evaluated by Chinese versions of the 36-item Short-Form (SF-36) Health Survey [23]. The SF-36 includes one multi-item scale that assesses eight health concepts: Bodily Pain (BP), General Health (GH), Mental Health (MH), Physical Functioning (PF); Role Emotional (RE), Role Physical (RP), Social Functioning (SF) and Vitality (VT). The maximum score for each item is 100 (best possible health). The scores from all eight scales are combined to create two comprehensive indicators of physical and mental health: the physical component summary (PCS, including BP, GH, PF and RP) and the mental component summary (MCS, including MH, RE, SF and VT). Anxiety and depression symptoms were assessed by the validated Hospital Anxiety and Depression Scale (HADs) [24].

### Data analysis

The statistical analyses were performed by using the SPSS 22.0 for Windows statistical software package (SPSS Inc., Chicago, IL, USA). Comparisons of continuous variables with normal distribution were tested by using One-Way ANOVA, and the results were presented as mean ± SD. Multiple linear regressions were employed to analyze the effects of overlapping FD, BD and FH on the HAD and SF-36 continuous variables. Regression coefficient and 95% confidence intervals (CI) were calculated. All statistical tests were two-tailed and a *P* value < 0.05 was deemed to be statistically significant.

## Results

### Study population

IBS was diagnosed in 319 patients according to Rome III criteria and completed the questionnaires. The ratio of female/male was 1.17:1 and the mean age was 41.4 ± 13.2 years old. Based on the Rome III criteria, IBS-D is the most common subtype, accounting for 67% of the patients, followed by IBS-C (16%), IBS-U (12%) and IBS-M (5%). The prevalence of FD, BD and FH in IBS patients was 40.13, 29.47 and 11.29% respectively. Of the 319 patients with IBS, 169 (52.98%) patients had overlapping upper FGIDs, among whom 95(29.78%) had two overlapping conditions, 59 (18.50%) had three overlapping conditions and 15 (4.70%) had four overlapping conditions (Table 1).

**Table 1:** Psychological factor and health-related quality of life among different overlapping conditions

	N	Anxiety	Depression	MCS	PCS	SF-36
IBS only	150	8.31 ± 3.09	6.15 ± 3.62	61.47 ± 21.38	63.73 ± 16.22	62.60 ± 17.16
Two overlapping conditions	95	8.73 ± 3.66	6.69 ± 4.08	55.41 ± 23.93^a^	57.67 ± 18.09^a^	56.54 ± 19.56^a^
Three overlapping conditions	59	8.71 ± 3.40	7.17 ± 3.33	52.09 ± 20.68^a^	54.97 ± 17.32^a^	53.53 ± 17.58^a^
Four overlapping conditions	15	7.60 ± 3.80	6.40 ± 3.87	47.91 ± 24.94^a^	49.27 ± 21.23^a^	48.59 ± 21.84^a^

^a^Compared with IBS only, *P* < 0.05

*Abbreviations*: *IBS* irritable bowel syndrome, *MCS* mental component summary, *PCS* physical component summary, *SF-36*, 36-item Short-Form Health Survey

### Relationship between the number of coexisting FGIDs and HAD and HRQoL

There were no significant differences in anxiety and depression scores between the four groups according to the number of coexisting FGIDs (Table 1). The scores of MCS, PCS and SF-36 in patients with overlapping conditions were significantly lower than those with IBS only (*P* < 0.05). When examining the associations between the number of coexisting FGIDs and HRQoL, we found that there were no significant differences between two, three and four overlapping conditions (*P* > 0.05).

### The impact of overlapping FD, BD and FH on scores of anxiety and depression

To analyze the impact of concomitant FD, BD and FH, the IBS patients were categorized into IBS + FD, IBS + FD + BD/FH (BD and/or FH), IBS + BD/FH and IBS only. The anxiety and depression scores in IBS + FD seemed to be the highest among the four groups. The IBS + FD group had a statistically significant higher anxiety score than the IBS + BD/FH group (*P* < 0.05), and also a statistically significant greater impact on depression compared with the IBS only group (Table 2, *P* < 0.05). The latter observation remained consistent after adjustment for confounding variables such as age and gender (*P* < 0.05, Table 3).

**Table 2:** Psychological factor and health-related quality of life according to the symptom overlap of FD, BD and FH in IBS patients

subgroup	N	Anxiety	Depression	MCS	PCS	SF-36
IBS only	150	8.31 ± 3.09	6.15 ± 3.62	61.47 ± 21.38	63.73 ± 16.22	62.60 ± 17.16
IBS + FD	61	9.21 ± 3.70	7.44 ± 4.05^a^	52.02 ± 23.63^a^	55.48 ± 17.88^a^	53.75 ± 19.15^a^
IBS + BD/FH	41	7.76 ± 3.34^b^	6.10 ± 4.19	59.17 ± 23.54	61.24 ± 18.39^c^	60.20 ± 19.67^c^
IBS + FD + BD/FH	67	8.61 ± 3.55	6.73 ± 3.26	51.59 ± 21.65^a^	53.21 ± 17.87^a^	52.40 ± 18.42^a^
IBS only	150	8.31 ± 3.09	6.15 ± 3.62	61.47 ± 21.38	63.73 ± 16.22	62.60 ± 17.16
IBS + BD	26	7.73 ± 3.67	5.85 ± 3.94	61.91 ± 23.56	61.18 ± 17.95	61.54 ± 19.47^d^
IBS + FD/FH	75	9.04 ± 3.53	7.07 ± 3.97	52.09 ± 23.93^a^	55.89 ± 18.09^a^	53.99 ± 19.53^a^
IBS + BD + FD/FH	68	8.50 ± 3.57	6.96 ± 3.56	52.05 ± 21.12^a^	54.09 ± 18.22^a^	53.07 ± 18.27^a^
IBS only	150	8.31 ± 3.09	6.15 ± 3.62	61.47 ± 21.38	63.73 ± 16.22	62.60 ± 17.16
IBS + FH	8	8.25 ± 2.87	3.75 ± 3.28	60.17 ± 25.30	62.91 ± 19.56	61.54 ± 21.21
IBS + FD/BD	133	8.84 ± 3.66	6.88 ± 3.79^e^	54.65 ± 22.55^a^	56.37 ± 17.53^a^	55.51 ± 18.56^a^
IBS + FH + FD/BD	28	7.68 ± 3.27	7.50 ± 3.74^e^	46.65 ± 23.45^a^	52.13 ± 20.53^a^	49.39 ± 20.77^a^

^a^Compared with IBS only, *P* < 0.05

^b^compared with IBS + FD, *P* < 0.05

^c^compared with IBS + FD + BD/FH, *P* < 0.05

^d^compared with IBS + BD + FD/FH, *P* < 0.05

^e^compared with IBS + FH, *P* < 0.05

*Abbreviations*: *IBS* irritable bowel syndrome, *FD* functional dyspepsia, *BD* belching disorders, *FH* Functional heartburn, *BD/FH* BD and/or FH, *FD/FH* FD and/or FH, *FD/BD* FD and/or BD, *MCS* mental component summary, *PCS* physical component summary, *SF-36* 36-item Short-Form Health Survey

**Table 3:** Multivariate analysis on factors that influenced anxiety and depression scores of patients with irritable bowel syndrome

	Anxiety		Depression	
	Regression Coefficient(95%CI)	*P* value	Regression Coefficient(95%CI)	*P* value
Male	reference		reference	
Female	0.241(− 0.530, 1.012)	0.539	0.673(− 0.184, 1.529)	0.123
Age	− 0.026(− 0.054, 0.003)	0.084	− 0.007(− 0.039, 0.024)	0.644
IBS overlap				
IBS only	reference		reference	
IBS + FD	0.929(−0.071, 1.929)	0.069	1.245(0.134, 2.357)	0.028
IBS + FD + BD/FH	0.345(−0.652, 1.342)	0.497	0.407(−0.702, 1.515)	0.471
IBS + BD/FH	−0.532(−1.690, 0.625)	0.366	− 0.069(− 1.356, 1.218)	0.916

*Abbreviations*: *IBS* irritable bowel syndrome, *FD* functional dyspepsia, *BD* belching disorders, *FH* Functional heartburn, *BD/FH* BD and/or FH, *IBS-U* unsubtyped IBS, *IBS-D* IBS with diarrhea, *IBS-C* IBS with constipation, *IBS-M* mixed IBS

Furthermore, the IBS patients were subgrouped into IBS + BD, IBS + FD/FH (FD and/or FH), IBS + BD + FD/FH and IBS only according to the overlap of BD. There was not statistical difference between IBS only and IBS + BD in anxiety and depression score (Table 2, *P* > 0.05). Similarly, no significant difference was observed between IBS only and IBS + FH in anxiety and depression (Table 2, *P* > 0.05). The depression score was significantly lower in IBS + FH group, compared with those in IBS + FD/BD (FD and/or BD) and IBS + FH + FD/BD (*P* < 0.05). Given the limited impact of concomitant BD and FH on anxiety and depression for a univariate analysis, the multivariate analysis was not specifically performed.

### The impact of overlapping FD, BD and FH on HRQoL of IBS patients

In the univariate analysis, MCS, PCS and SF-36 were significantly lower in IBS + FD and IBS + FD + BD/FH groups compared with those in the IBS only group (Table 2, *P* < 0.05). In addition, IBS + FD + BD/FH had a more significant impact on PCS and SF-36 compared with IBS + BD/FH (*P* < 0.05).

**Fig. 1:**
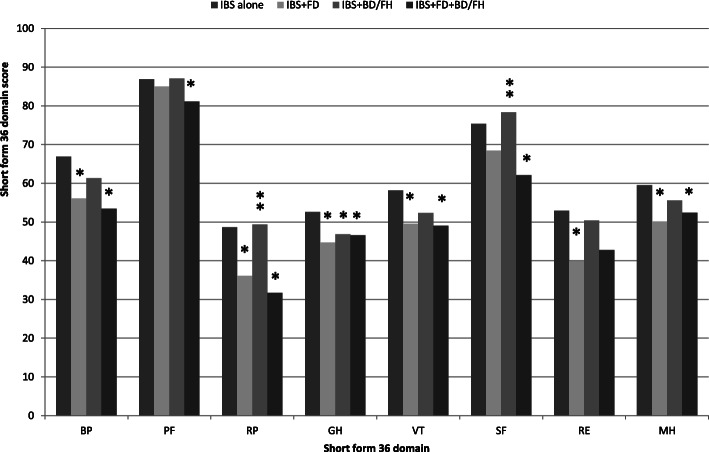
Means of SF-36 dimensions according to the symptom overlap in IBS patients. *compared with IBS only, *P* < 0.05; ** compared with IBS + FD + BD/FH, *P* < 0.05. BP, Bodily Pain; PF, Physical Functioning; RP, Role Physical; GH, General Health; VT, Vitality; SF, Social Functioning; RE, Role Emotional; MH, Mental Health

The IBS + FD + BD/FH group reported significantly poorer HRQoL assessed through the SF-36 in all dimensions except for RE, as compared to the IBS only group (*P* < 0.05). IBS + FD presented significantly lower scores in most dimensions except in the SF and PF compared with IBS only (*P* < 0.05). However, no statistically significant difference was seen between the IBS + FD and the IBS + FD + BD/FH group (*P* > 0.05). The scores of RP and SF were significantly lower in the IBS + FD + BD/FH group compared with those in the IBS + BD/FH group (*P* < 0.05). Subjects with IBS + BD/FH had a statistically significant impairment of HRQoL in GH, when compared to those with IBS only (*P* < 0.05, Fig. 1).

We also observed that there were no significant differences between IBS only and IBS + BD as well as between IBS only and IBS + FH in the scores for MCS, PCS and SF-36 (Table 2, *P* > 0.05). IBS + FD/FH, IBS + BD + FD/FH, IBS + FD/BD, IBS + FH + FD/BD had worse MCS, PCS and SF-36 scores when compared with IBS only(*P* < 0.05). Additionally, IBS + BD had significantly higher SF-36 score than IBS + BD + FD/FH (*P* < 0.05).

Multiple linear regression analysis showed that poorer SF-36 global health was predicted by IBS + FD and IBS + FD + BD/FH independent of age, gender, anxiety and depression as compared to IBS only (*P* < 0.05). IBS + FD + BD/FH was a predictive factor of lower MCS and PCS scores while IBS + FD was a predictor of poorer PCS compared with IBS only (*P* < 0.05, Table 4). Similarly, given the limited impact of concomitant BD and FH on PCS, MCS and SF-36 for a univariate analysis, the multivariate analysis was not specifically performed.

**Table 4:** Multivariate analysis on factors that influenced health-related quality of life in patients with irritable bowel syndrome

	MCS		PCS		SF-36 global health	
	Regression Coefficient (95%CI)	*P* value	Regression Coefficient(95%CI)	*P* value	Regression Coefficient(95%CI)	*P* value
Male	reference		reference		reference	
Female	2.259(−2.059,6.402)	0.281	−0.112(−3.785,3.560)	0.952	1.073(−2.446,4.593)	0.549
Age	0.036(−0.106,0.199)	0.641	−0.078(− 0.214,0.058)	0.260	− 0.021(− 0.151,0.109)	0.752
Anxiety	−1.392(−2.046,-0.684)	< 0.001	− 0.475(− 1.084,0.134)	0.126	−0.933(− 1.517,-0.350)	0.002
Depression	− 2.829(− 3.398,-2.160)	< 0.001	− 1.595(− 2.143,-1.047)	< 0.001	−2.212(− 2.737,-1.687)	< 0.001
IBS overlap						
IBS only	reference		reference		reference	
IBS + FD	−4.818(−10.183,0.547)	0.078	−5.594(− 10.383,-0.805)	0.022	−5.206(−9.796,-0.616)	0.026
IBS + FD + BD/FH	−8.697(−14.005,-3.390)	0.001	−9.041(−13.779,-4.302)	< 0.001	−8.869(− 13.410,-4.328)	< 0.001
IBS + BD/FH	−3.356(− 9.521,2.809)	0.285	−2.735(− 8.238,2.769)	0.329	− 3.046(− 8.320,2.229)	0.257

*Abbreviations*: *MCS* mental component summary, *PCS* physical component summary, *SF-36* 36-item Short-Form Health Survey, *IBS* irritable bowel syndrome, *FD* functional dyspepsia, *BD* belching disorders, *FH* Functional heartburn, *BD/FH* BD and/or FH, *IBS-U* unsubtyped IBS, *IBS-D* IBS with diarrhea, *IBS-C* IBS with constipation, *IBS-M* mixed IBS

## Discussion

Until now, most of studies relevant to the co-existence of IBS and upper FGIDs were focused on the overlap between IBS and FD. Coexistence with FD is known to exert deleterious effects on HRQoL and psychological status of IBS patients [11, 22]. The impact of other upper FGIDs on patients with IBS, however, has not been examined. Thus, to the best of our knowledge, this is the first prospective study in which the impact of concomitant FD, BD and FH on anxiety, depression and HRQoL in patients with IBS has ever been evaluated. In the present study, we found that the IBS + FD group had a statistically significant impairment in anxiety, depression and PCS. Furthermore, IBS + FD + BD/FH is a predictive factor of reduced MCS, PCS and SF-36 scores in IBS subjects. The IBS + FD and IBS + FD + BD/FH groups had a statistically significant impairment in most of SF-36 dimensions, while the IBS + BD/FH group reported poorer HRQoL only in GH as compared to the IBS only group. The impact of concomitant BD and FH on the psychological status and HRQoL of IBS patients was limited.

The concomitant BD, FH and FD in IBS patients had multiple overlapping combinations, which made it difficult to analyze the impact of each individual upper FGID. First of all, we compared the anxiety, depression and HRQoL scores between the four groups according to the number of coexisting FGIDs. We found that the scores of MCS, PCS and SF-36 in the overlapping conditions were significantly lower than those in the IBS only, and that there were no significant differences in the anxiety and depression scores between the four groups. Furthermore, no significant differences in HRQoL were observed between two, three and four overlapping conditions. The results indicated that the psychological scores and HRQoL in IBS patients were not associated with the number of coexisting upper FGIDs. It suggested that the impact of coexisting BD, FD and FH on psychological scores and HRQoL can be analyzed according to the concomitant upper FGIDs, without having to consider the number of these upper FGIDs. Our findings were not in line with the recently published data by Pinto-Sanchez MI et al [8] which indicated that the prevalence of anxiety and depression increased in a stepwise manner with the number of coexisting FGIDs among the outpatients. The difference in results may be because our study focused on three upper FGIDs while the investigation by Pinto-Sanchez MI et al. included far more FGIDs.

Lee HJ reported that depressive mood was significantly related to IBS + FD overlap rather than IBS alone based on the Rome III criteria [11]. Work by Pinto-Sanchez MI et al has demonstrated that IBS + FD and IBS + FH can result in an increased risk in anxiety (OR = 3.78, OR = 1.85 respectively) and depression (OR = 4.80, OR = 2.11 respectively) compared with IBS only [8]. Notably, the coexistence of BD or FH in the IBS + FD and concomitant FD or BD in the IBS + FH patients has not been distinguished by these studies. Consistent with other studies, we observed the depression score in IBS + FD was significantly higher as compared to IBS only. Moreover, the anxiety score of the IBS + FD group was significantly higher than that of the IBS + BD/FH group. There were no significant differences in anxiety and depression between the individuals with IBS + FD and those with IBS + FD + BD/FH. Furthermore, no significant difference was observed between IBS only and IBS + BD as well as between IBS only and IBS + FH in the scores for anxiety and depression. Psychiatric comorbidities modified the experience of illness and illness behavior such as health care seeking and contributed to poor outcomes [21, 25]. These factors can be reduced or buffered by adaptive coping skills, social support, cognitive behavioral therapy and medication treatment [25, 26]. Awareness of identifying and resolving these associated manifestations will likely assist in the development of more effective diagnostic and treatment strategies for IBS + FD patients. The evidence of lower score of anxiety and depression in the IBS + BD/FH group and of no significant difference between the IBS + FD and IBS + FD + BD/FH groups suggested the impact of concomitant BD and FH on psychiatric status was not as serious as that of IBS + FD, and more studies are needed to understand and confirm these findings.

Patients with supragastric belching reported a decreased HRQoL in dimensions of SF, MH, VT, BP and GH but not for PF, RP and RE [27]. Lower HRQoL was also observed in IBS patients with heartburn compared with those without heartburn [28]. The limitation of the latter study was that the heartburn was symptom-based, neither defined by FH nor by gastroesophageal reflux disease (GERD). To our knowledge, the impact of coexisting BD and FH on the HRQoL of IBS patients has not been studied in detail. We further evaluated the impact of overlapping FD, BD and FH on SF-36 in the present study, and found that the IBS patients with overlapping FD and FD + BD/FH had lower SF-36 scores than those with IBS only. Multiple linear regression analysis showed that IBS + FD + BD/FH was a predictor of poorer MCS and PCS and IBS + FD was a predictor of poorer PCS as compared to the IBS only. In addition, IBS + FD and IBS + FD + BD/FH group had a statistically significant impairment in most of SF-36 dimensions. For subjects with IBS + BD/FH, only the score of GH was statistically significant lower compared to that of IBS only whereas no difference was seen between IBS + BD/FH and IBS only in the other dimensions. Therefore, IBS patients with FD or FD + BD/FH overlap should be a focus of attention due to their greater detrimental effect on quality of life.

Our study has some potential limitations. First, the diagnosis of FH was based on questionnaire and on Rome III criteria, without excluding GERD by ambulatory pH monitoring, proton pump inhibitor trials and endoscopy; second, the coexistence of BD and FH has not been distinguished because of the limited cases in each symptom overlap; third, most of IBS patients in this study were IBS-D, which is different from the subtype distribution in western countries, hence, the conclusion may not be applicable to western population; fourth, we did not analyze other factors known to affect the HRQoL in patients with IBS such as somatic comorbidities and symptom severity - which may have confounded our results.

## Conclusions

Among the diarrhoea-prevalent IBS patients, patients presenting with concomitant IBS and upper FGIDs report poorer MCS, PCS and SF-36 compared with those with IBS only. Patients with overlapping FD and IBS experience more anxiety, depression and lower PCS. The coexisting FD + BD/FH is a predictive factor of reduced MCS and PCS in IBS patients. The impact of concomitant BD and FH on psychological status and HRQoL of IBS patients was limited. These findings implicated that the coexisting upper FGIDs might be treated distinctively according to their impact on the psychological status and HRQoL when developing comprehensive management strategies for IBS treatment.

## Data Availability

The datasets used and analyzed in the current study are available from the corresponding authors upon request.

## Abbreviations

BD: Belching disorders
BP: Bodily pain
CI: Confidence intervals
FD: Functional dyspepsia
FH: Functional heartburn
FGIDs: Functional gastrointestinal disorders
GH: General Health
HADs: Hospital Anxiety and Depression scale
HRQoL: Health-related quality of life
MCS: The mental component summary
MH: Mental Health
IBS: Irritable bowel syndrome
IBS-C: IBS with constipation
IBS-D: IBS with diarrhea
IBS-M: Mixed IBS
IBS-U: Un-subtyped IBS
PCS: Physical component summary
PF: Physical functioning
RE: Role emotional
RP: Role physical
SF: Social functioning
SF-36: Short-Form Health Survey
VT: Vitality
